# Metabolic Phenotyping of Nutritional Rickets in Bangladeshi Children

**DOI:** 10.3390/nu18101580

**Published:** 2026-05-15

**Authors:** Elizabeth A. Wimborne, Sonia Ahmed, Kate A. Ward, Ann Prentice, John M. Pettifor, Rubhana Raqib, Swapan Kumar Roy, Shahidul Haque, Jonathan R. Swann

**Affiliations:** 1 School of Human Development and Health, Faculty of Medicine, University of Southampton, Southampton SO16 7QF, UK; e.wimborne@soton.ac.uk (E.A.W.);; 2 MRC Human Nutrition Research, Elsie Widdowson Laboratory (Closed December 2018), Cambridge CB1 9NL, UKannp@mrclmb.ac.uk (A.P.); 3 School of Health & Life Sciences, Teeside University, Middlesbrough TS1 3BX, UK; 4 Institute of Metabolic Science (IMS) Epidemiology, University of Cambridge, Cambridge CB2 0QQ, UK; 5 SAMRC/Wits Developmental Pathways for Health Research Unit, Department of Paediatrics, University of the Witwatersrand, Johannesburg 2050, South Africa; john.pettifor@wits.ac.za; 6 International Centre for Diarrhoeal Disease and Research (icddr,b), Dhaka 1000, Bangladesh; 7 Bangladesh Breastfeeding Foundation, Dhaka 1206, Bangladesh; 8 Social Assistance for Physically Vulnerable People (SARPV), Dhaka 1216, Bangladesh

**Keywords:** metabolomics, nutritional rickets, calcium deficiency

## Abstract

**Background/Objectives**: Nutritional rickets is a childhood bone disorder leading to skeletal deformities and life-long disabilities. Early-stage diagnosis remains challenging due to the limited availability of non-invasive tools. This study explores metabolic variation associated with the active disease stages and with etiological factors, such as nutritional deficiencies and biochemical alterations. **Methods**: Untargeted ^1^H NMR spectroscopy-based metabolomics were performed on urine and plasma samples collected from Bangladeshi children with radiologically active rickets (AR; *n* = 24; aged 2.98 ± 1.19 years), inactive rickets (IR; *n* = 36; aged 3.39 ± 1.87 years), and healthy matched controls (*n* = 58; aged 3.58 ± 1.59 years). This analysis also integrated corresponding clinical biochemistry and dietary intake data previously collected from the cohort. **Results**: Orthogonal Partial Least Squares-Discriminant Analysis (OPLS-DA) identified the 24 h urinary excretion of 13 metabolites to vary with AR, including those previously associated with bone metabolism such as β-aminoisobutyrate, *N*-methylnicotinamide, taurine and hypoxanthine. Biochemically, AR was strongly characterized by increased plasma alkaline phosphatase and decreased iFGF23. The multi-block integration of metabolomic, biochemical, and nutritional data achieved an 18.6% classification error rate. Children with IR exhibited metabolic profiles similar to healthy controls, aligning with their clinical resolution. **Conclusions**: Active nutritional rickets presents a distinct metabolic profile, highlighting novel biologically relevant metabolites. These exploratory signals provide insights into the physiological impact of the disease and warrant further targeted investigation to assess their potential for informing early non-invasive detection and preventive interventions. In the long term, such tools are vital to prevent irreversible skeletal damage and to help mitigate lifelong physical disability and the resulting social vulnerability for affected children.

## 1. Introduction

Micronutrient deficiencies, often termed hidden hunger, can result in adverse developmental outcomes for children, despite individuals receiving sufficient calorific energy. Nutritional rickets, a bone disorder in growing children, represents a childhood micronutrient deficiency and a prevalent global health concern. Studies conducted in countries with unlimited exposure to sunlight, such as South Africa [1], the Gambia [2,3], Nigeria [4], Malawi [5], and Bangladesh [6], have indicated the role of chronically low dietary calcium intake in the development of this condition. In Chakaria alone, a south-eastern region of Bangladesh, nutritional rickets affects approximately 50,000 individuals [7].

This condition is typified by poor mineralization at the growth plate, where long bones normally elongate through the proliferation and hypertrophy of chondrocytes, followed by vascularization and calcification [8,9]. In nutritional rickets, impaired apoptosis of these hypertrophic chondrocytes leads to their accumulation, hindering mineralization and disrupting the characteristic columnar arrangement of the growth plate [10]. Clinically, this manifests as swelling of the long bone ends, skeletal deformities, stunting, and bone pain [9,10]. If left untreated, long-term consequences include life-long disabilities, low bone density and mass, osteomalacia, and increased risk of obstructed labour due to pelvic narrowing [10].

Calcium, vitamin D and phosphorus are critical components involved in bone mineralization. Deficiencies in the intake of these factors, or disruption to their metabolism, are thought to drive this condition. Dietary calcium intake is below WHO recommendations in many regions globally [10], which is exacerbated by the reduced bioavailability of this nutrient with high dietary phytates or oxalates [11,12,13]. The body’s homeostatic response to low serum calcium involves an increase in parathyroid hormone (PTH), which maintains calcium levels at the expense of renal phosphate wasting, leading to hypophosphatemia [9,14]. Reduced phosphate abundance limits apoptosis in hypertrophic chondrocytes, leading to the characteristic clinical symptoms of rickets [9].

Diagnosis of nutritional rickets is typically made through the combinatory use of clinical, radiological, and biochemical criteria [15]. Available diagnostic techniques are often invasive, and methods relying on clinical symptoms or bone radiographic changes are only effective once growth restriction and bone deformities have already occurred following an extended period of nutritional deficiency due to compensatory mechanisms involved in calcium–phosphate–vitamin D metabolism [14,16]. As such, tools for the early diagnosis and detection of nutritional rickets are lacking, and this has been highlighted as a research gap by the WHO [10].

Biochemical changes associated with calcium deficiency have been explored in rodent urinary and plasma models and in the urinary metabolome of children with nutritional rickets [17,18,19]. These studies identified common predictive biomarkers, such as variations in indoxyl sulphate, phosphate, and taurine. However, no study so far has investigated this in the plasma metabolome of children with nutritional rickets in geographical regions outside China [19]. As the etiology of nutritional rickets has not been fully agreed upon, understanding the metabolic consequences of different causal factors or geographical presentations of nutritional rickets is essential in aiding the diagnosis and treatment of this condition.

This study aimed to investigate the biochemical implications of nutritional rickets in early life. To achieve this, the urinary and plasma metabolomes of children living in Chakaria, Bangladesh, were studied. Previously, an association between rickets and low dietary calcium intake was identified in this cohort, alongside associations with exposure to metals and low vitamin D status [6]. Developing a greater understanding of the widespread metabolic derangements associated with nutritional rickets will aid in the understanding of the disease physiology, which could inform future research into the development of early, non-invasive diagnostic tools.

## 2. Materials and Methods

### 2.1. Study Design

This study is a secondary analysis of a cohort who were investigated to determine the etiology of nutritional rickets in Bangladesh. The detailed study design, cohort descriptions, methods of data collection, and primary outcomes have been published previously [6]. This study was approved by the Research and Ethics Review Committees of the International Centre for Diarrhoeal Disease and Research, Bangladesh (ICDDR, B; 31 March 2011), and by the University of Southampton Research Ethics Committee (reference number 64235; 31 July 2022).

### 2.2. Study Setting

This study was based in south-eastern Bangladesh in the coastal Cox’s Bazar subdistrict of Chakaria from August 2011 to January 2012. This region had the highest prevalence of rickets (2.19%) of all six regions of Bangladesh in the 2008 Bangladesh national rickets survey of children aged 1–15 years [7]. At the time of the study, the Chakaria Health and Demographic Survey 2012 reported that menial labour made up around 50% of household incomes in the area, including activities such as agricultural farming, woodcutting, and fishing [20]. There were also poor sanitation levels, with only 50% of households having access to a sanitary latrine [20]. Chakaria experiences a tropical climate, with an average annual temperature of 25 °C (varying between 19 °C and 28 °C), and an average annual rainfall of 335 cm, predominantly falling during the rainy season (April–September) [20]. A large proportion of the population is Muslim (>90%), with smaller populations of Hindus and Buddhists [20]. Typical clothing for children in the area includes short-sleeved shirts with skirts/shorts above the knee, with no head covering or hijab [7]. The diet is predominantly rice- and plant-based, with a low calcium content, and rich in phytates and oxalates [21,22,23].

### 2.3. Participants

Children aged one to 10.9 years of age were recruited from the Social Assistance and Rehabilitation for the Physically Vulnerable (SARPV, http://sarpv.org/; accessed on 7 May 2026) rickets clinic in Chakaria. The recruitment criteria included this being their first presentation with limb deformities at the clinic and receiving a diagnosis of rickets according to the SARPV clinic criteria. This diagnosis involved the presence of rachitic signs on radiographs of the knees and wrists, as well as a physical examination on the nature and severity of the deformity. A definitive, independent assessment conducted after fieldwork had finished confirmed the diagnosis using the ten-point radiographic Thacher Scoring Method, which is widely used clinically [24]. The Thacher Scoring Method grades radiographic images of both wrists and knees on the presence and extent of cupping and fraying of the growth plates. In addition, plasma total alkaline phosphatase (ALPTot) measures were available from biochemical analyses to further indicate the presence of active disease [25].

From these findings, the children were stratified into those with a presentation of active rickets (AR; total score of ≥1.5 on the Thacher Scoring system) and those with bone deformities but no evidence of active disease, defined as having an inactive disease presentation (IR). This latter group presented with observable bone deformities; however, they had a ALPTot of less than 300 U/L and/or a Thacher score of <1.5. Exclusion criteria for the study included taking medication or nutrient supplements that might interfere with bone metabolism, individuals with intestinal or renal disease, or any other condition causing physical disability and impaired mobility. A local healthy control child, who was recruited for each rickets case (active or inactive) using the Chakaria demographic surveillance survey, was matched for sex, village, and as closely as possible for age [26]. These children had no observable limb deformities. Written consent was obtained from the child’s parent or guardian following an explanation in their native language.

In the full cohort [6], 24 children had AR (median Thacher score = 6 [25th percentile = 3; 75th percentile = 10]), and 38 had IR (median Thacher score = 0 [25th percentile = 0; 75th percentile = 0]). The age of participants ranged from 1.0 to 10.6 years and children with AR and IR were on average younger than their respective controls (AR median age (years) 2.7 [25th percentile = 2.1, 75th percentile = 3.4; AR controls = 3.2 [2.5, 4.3]; IR = 3.0 [1.9, 4.3], IR controls = 3.2 [2.4, 5.1]). There were equal numbers of males and females in the children with AR and their controls (12M and 12F in each group), but there were more males than females with IR and their controls (28M and 10F in each group). Dietary information was collected by a 24 h direct weighing of all foods and drinks consumed, together with structured questionnaires covering the frequency of consumption of calcium-rich foods consumed over the past year, and retrospective information on infant feeding practice. Nutrient intakes were calculated using a bespoke in-house dietary assessment platform developed at MRC Human Nutrition Research, Cambridge, UK, using published composition data on Bangladeshi and Indian foods [6]. All the children had been breast-fed in infancy. At the time of the study, most of the children (82%) were fully weaned; the remaining children were receiving some breast-milk as part of a mixed diet (AR = 4; AR controls = 7; IR = 9; IR controls = 2). Participants who provided biological samples (stored) for metabolomics were included in this secondary analysis; the data on anthropometry, dietary intake and biochemistry for these children were obtained from the full cohort database and the methods described by Ahmed et al. [6].

### 2.4. Sample Collection

A timed two-hour urine sample was collected at the SARPV clinic after an overnight fast. A 24 h urine sample was collected on a separate day during a home visit. Both urine collections were started in the early morning with the second void of the day, the first having been discarded. Urine was collected directly into urine bags (Romsons, Paediatric Urine Collection Bag, DB 1062, New Delhi, India) and then poured into acid-washed containers. The urine samples were initially refrigerated or chilled on ice in an insulated box, then total volumes were measured, and aliquots were frozen at −20 °C. A venous blood sample was taken at one hour into the two-hour urine collection and placed on ice. Within one hour of collection, plasma was separated in a refrigerated centrifuge at 3000 rpm for 15 min, aliquoted, and then frozen. This involved temporary storage at −20 °C in the clinic, before transportation on dry ice and storage at the ICDDRB in Dhaka, Bangladesh, at −80 °C. Following study completion, frozen samples were shipped on dry ice to MRC Human Nutrition Research, Cambridge, UK, for initial biochemical and immunoassay analysis [6]. The residual samples were then shipped on dry ice to University of Southampton, UK, for metabolomic analysis.

### 2.5. Analysis of Samples

#### 2.5.1. ^1^H Nuclear Magnetic Resonance (NMR) Spectroscopy

Metabolic profiles of the urine (non-acidified aliquots) and plasma were measured by untargeted ^1^H NMR spectroscopy. For the urinary samples, 60 μL of phosphate-buffered solution (pH 7.4, 100% D_2_O) containing 1 mM of the internal standard 3-trimethylsilyl-one-[2,2,3,3-2H4] propionate (TSP) was added to 540 μL of urine, vortexed and span at 10,000× *g* for 10 min at 4 °C. These samples were transferred to 5 mm NMR tubes. Plasma samples were prepared by centrifuging 100 μL of thawed plasma at 10,000× *g* for 10 min through ultra −0.5 mL central filter units which had undergone seven washes with distilled water by centrifugation before sample addition. The resulting plasma was combined with 100 μL of potassium buffer solution (pH 7.4, 100% D_2_O) 1/10 diluted with D_2_O containing 1 mM of TSP. Samples were vortexed to mix spun at 10,000× *g* and the supernatant was transferred to 3 mm NMR tubes. To monitor variability across the sample run, a pooled quality control urine or plasma sample (containing 5 μL of each sample) was added to each plate.

All samples were measured with a 700 MHz Bruker NMR spectrometer equipped with a cryoprobe and refrigerated SampleJet autosampler maintained at 6 °C (Bruker Biospin GmbH, Rheinstetten, Germany). A standard one-dimensional solvent suppression pulse sequence (relaxation delay, 90° pulse, 4 ms delay, 90° pulse, mixing time, 90° pulse, acquire free induction decay) was used to measure each sample. Each spectrum was acquired with 32 scans, four dummy scans, 64,000 frequency domain points and a spectral window set to 20 ppm (parts per million). All spectra were automatically phase- and baseline corrected and referenced to the TSP resonance at δ 0.0 in Topspin 3.2 (Bruker Biospin GmbH, Rheinstetten, Germany). Raw spectra were digitized, aligned, and normalized in Matlab (version 2018a, MathWorks Inc., Natick, MA, USA) using the Imperial Metabolic Profiling and Chemometrics Toolbox (https://github.com/csmsoftware/IMPaCTS; accessed 7 May 2026). Redundant spectral peaks (TSP, water, and urea) were excised, and the resulting spectra were manually aligned using a recursive segment-wise peak alignment method. A probabilistic quotient normalization method was used to reduce the effects of variation in sample dilution. NMR spectral peaks provide a measure of the relative abundance of the metabolite giving rise to that signal. Urinary metabolite peaks associated with AR were integrated and log transformed for downstream analysis.

**Figure 1:**
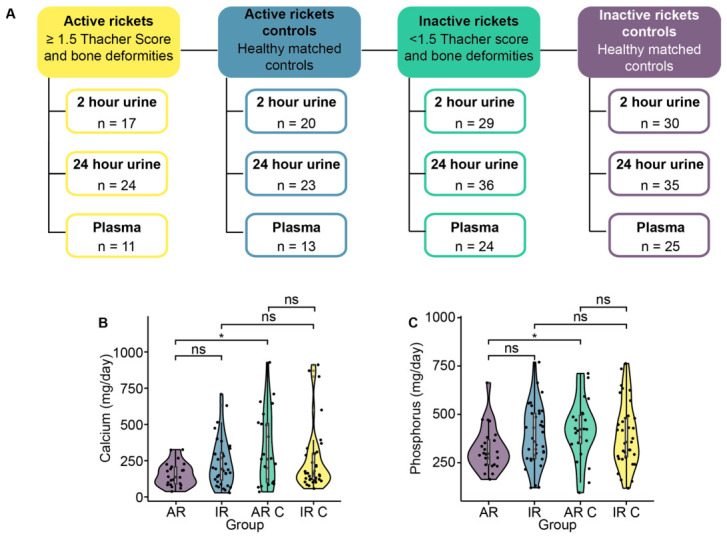
Study design. (**A**) Sample numbers for metabolomic analysis per group. The daily intake of (**B**) calcium and (**C**) phosphorus (mg/day) for children in the active rickets (AR; *n* = 24), inactive rickets (IR; *n* = 36), active rickets control (AR C; *n* = 23), and inactive rickets control (IR C; *n* = 35) groups. Significance determined by *t* test. * = *p* < 0.05; ns = *p* > 0.05.

Principal Component Analysis (PCA) models were used to perform quality control checks on the spectra, including an assessment of biological QC samples, outliers, and batch effects. One urinary ^1^H NMR spectrum, and 23 plasma ^1^H NMR spectra were removed. The large number of plasma samples excluded was due to poor spectra arising from low sample concentrations. Final sample numbers are documented in Figure 1A.

#### 2.5.2. Biochemical Measurements

Heparinized-plasma samples were used for the analysis of all plasma analytes except for intact parathyroid hormone (PTH) and C-terminal fibroblast growth factor 23 (cFGF23), which were measured in EDTA samples. Intact PTH was quantified using an immunometric assay on the Immulite 1000 chemiluminescence analyzer (Siemens Health Care, Diagnostics Products Ltd., Gwynedd, UK). Chemiluminescence immunoassays (Liaison; DiaSorin, Stillwater, MN, USA) were used to measure plasma 25-hydroxyvitamin D (25OHD) and bone alkaline phosphatase (BALP), while plasma 1,25-dihydroxyvitamin D (1,25(OH)_2_D) was assessed by radioimmunoassay (IDS Ltd., Boldon, Tyne and Wear, UK). Measurement of intact FGF23 (iFGF23) was conducted at VU University Medical Center, Amsterdam, using the Kainos ELISA kit (Tokyo, Japan). For cFGF23, a second-generation C-terminal two-site enzyme-linked immunosorbent assay (Immutopics Inc., San Clemente, CA, USA) was employed. The detection threshold for iFGF23 was set at 5 pg/mL, and concentrations below this limit were assigned a nominal value of 2.5 pg/mL.

Other plasma analytes were measured on the Dimension Xpand Clinical Chemistry System (Siemens Healthcare Diagnostics, Camberley, UK) using colorimetric methods. Total alkaline phosphatase (TALP), calcium (Ca), phosphate (Phos), magnesium (Mg), zinc (Zn), and creatinine (Cr) were measured in heparinized plasma samples. Additional plasma markers, including albumin, bilirubin, aspartate transaminase (AST), and ferritin, were measured via automated immunoassays. C-reactive protein (CRP) and α1-acid glycoprotein (AGP) were determined through particle-enhanced turbidimetric immunoassay using the Dimension Siemens ProSpec (Siemens, Munich, DE, USA).

All assays, aside from PTH, were conducted in duplicate to ensure accuracy. Performance was verified using both kit and in-house quality controls. Quality assurance for vitamin D measurements (25OHD and 1,25(OH)_2_D) was upheld as part of the Vitamin D External Quality Assessment Scheme (DEQAS, www.deqas.org, accessed 7 May 2026).

Prior to analysis, biochemical variables displaying positive skewness were log-transformed.

### 2.6. Data Analysis

#### 2.6.1. OPLS (Orthogonal Partial Least Squares) Models

Discriminant OPLS (OPLS-DA) models were built to identify urinary and plasma metabolic features or plasma biochemistry measures that distinguish between those with AR, IR, and their healthy controls. Additionally, OPLS regression models were constructed to identify urinary and plasma metabolic features that varied with continuous variables, such as anthropometry. The X matrix was composed of the ^1^H NMR spectra of the metabolome of interest or a dataset of clinical plasma biochemistry measures, whilst the variable of interest was used as the predictive Y component. For metabolomic analyses, OPLS models were built with MATLAB (version R2022b, MathWorks Inc.), while the biochemistry OPLS models were built using the *ropls* package (version 1.43.0; [27]) in R. Categorical Y variables were recoded to ones and zeros. To mitigate the risk of overfitting, a seven-fold cross-validation approach was used to assess the predictive capacity and stability of each model (Q^2^), whilst permutation testing evaluated model validity and statistical significance (1000 permutations). Models with Q^2^ > 0 and *p* < 0.05 were considered significant models. Discriminant metabolites associated with the variation between the groups were selected based on their covariance and the correlation profiles from the OPLS-DA models, and their spectral peaks were integrated. This integral was used as a quantitative measure of the relative abundance for the metabolite giving rise to that resonance.

#### 2.6.2. Data Integration Analysis for Biomarker Discovery Using Latent Components (DIABLO) Integration

DIABLO was performed to elucidate the relationships between biological datasets using the mixOmics package (version 6.34.0; [28]) in R. The model integrated datasets identified as differing between children with AR and controls, including urinary metabolites integrated from the ^1^H NMR spectra, nutritional intake, and plasma biochemistry, collected over 24 h. The optimal number of variables to retain for each component was determined using the tune function with seven-fold cross-validation to minimize the balanced error rate. To quantify the added value of data integration, the classification performance of the final DIABLO model was compared with the single-block Partial Least Squares Discriminant Analysis (PLS-DA) models constructed for each dataset individually. Relevance network analysis was performed to visualize pairwise correlations (*r* > 0.6) between selected features across the different data layers.

#### 2.6.3. Statistical Analysis

Metabolites identified as significantly contributing to the multivariate OPLS models were integrated to generate a relative abundance. Simple and adjusted linear regression models were created against the variable of interest using the relative abundance of the integrated metabolite. Adjusted linear regression models included the covariates age, sex, and WAZ. These analyses were performed using the *base stats* (version 3.6.2) package in R (version 4.4.0). To maintain statistical power, individuals were included in the analyses even if their matched sample was excluded due to low sample volume or quality control failure, and therefore the groups were treated as independent. Student’s T test and Wilcoxon rank sum tests were used to assess the differences between the anthropometric, nutritional, and demographic characteristics of the study groups for normally distributed and skewed data respectively. These tests were also used to compare characteristics of participants with included plasma NMR spectra compared to those with missing or excluded samples.

## 3. Results

### 3.1. Participant Characteristics

Of the 128 children recruited to the cohort [6], metabolic profiles were generated for 118 participants using ^1^H-NMR spectroscopy-based metabolomics (Figure 1A) performed on 24- (active rickets (AR) *n* = 24; AR controls *n* = 23; inactive rickets (IR) *n* = 36; IR controls *n* = 35) and two-hour (AR *n* = 17; AR controls *n* = 20; IR *n* = 29; IR controls *n* = 30) collected urine, as well as plasma samples (AR *n* = 11; AR controls *n* = 13; IR *n* = 24; IR controls *n* = 25). The anthropometric characteristics of the included individuals are detailed by group in Table 1, with full details of the whole cohort available in the work of Ahmed et al. [6]. Compared to their matched controls, the children with AR were lighter whilst also having shorter length, demi-span and sitting height. The children with IR also had shorter lengths than their matched controls but no differences in weight. When comparing between the rickets presentations, children with AR were also lighter and shorter than those with IR. No differences were observed between either case and control groups when considering other disease presentations (anemia, malaria, diarrhea, pneumonia, jaundice, dental abnormalities), birth order, family size, or sunscreen use. No differences in baseline demographic or anthropometric characteristics were observed between AR children with available plasma NMR spectra and those whose plasma samples were either missing or excluded following quality control (Supplementary Table S1). Within the remaining subgroups, participants with missing plasma data in the AR control group had greater WAZ (−1.69 ± 0.81 vs. −2.57 ± 1.15), those in the IR group had lower monthly income (3625 ± 1130 Taka vs. 5121 ± 3031 Taka), and those in the IR control group had a shorter demi-span (40.6 ± 3.28 cm vs. 44.0 ± 6.38 cm).

### 3.2. Nutritional Etiology of Nutritional Rickets

To confirm the contribution of nutritional intake to the pathophysiology of nutritional rickets, the daily intake of macro- and micro-nutrient intake was compared between children with AR, IR and matched controls using independent t-tests (Supplementary Table S2). Dietary calcium (AR, 156.5 ± 80.1 mg/d; controls, 329 ± 252.8 mg/d) and phosphorus (AR, 321.9 ± 112.5 mg/d; controls 415.8 ± 153.1 mg/d) intakes were significantly lower in the AR group compared to controls (Figure 1B,C). In contrast, no statistically significant differences were observed for other nutrients, or between any other study group.

### 3.3. Clinical Differences Between Active Rickets and Controls

**Figure 2:**
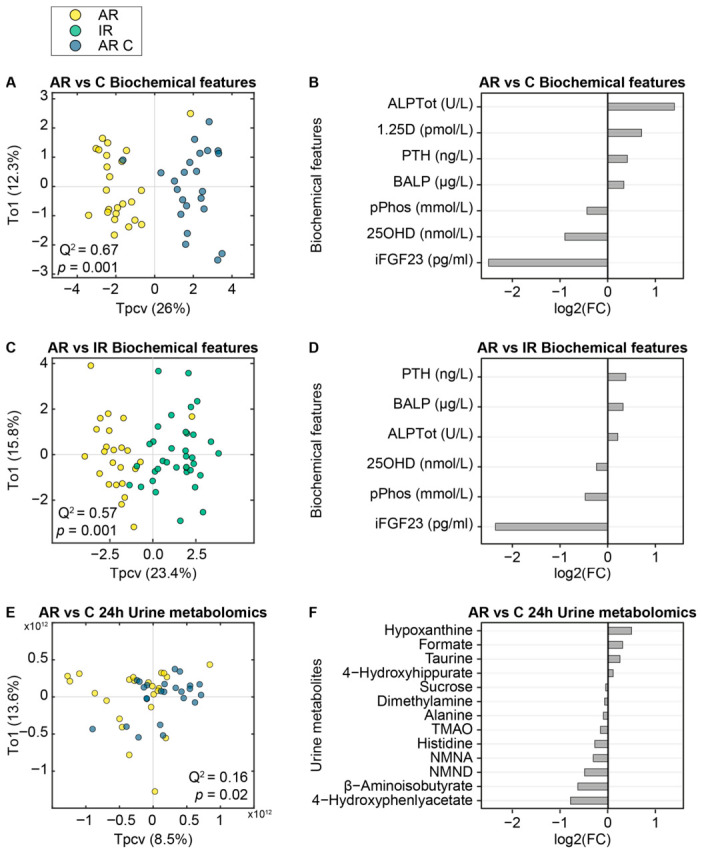
Children with active rickets (AR, yellow) are metabolically distinct from healthy matched controls (AR C, blue). (**A**) Multivariate Orthogonal Partial Least Squares–Discriminant Analysis (OPLS-DA) scores plot shows the separation of plasma biochemical profiles of children with AR and AR-C following seven-fold cross-validation and permutation testing. The Q^2^ value (0.67) indicates the predictive capacity of the model. The *x*-axis (*Tpcv*) represents the predictive variation between groups; the *y*-axis (*To1*) represents variation unrelated to group separation. Points represent individual participants. (**B**) Bar chart of log2(fold change) of plasma biochemical features identified as important in the OPLS-DA model classifying children with AR and AR C children. (**C**) OPLS-DA cross-validated scores plot the plasma biochemical features of children with AR and inactive rickets (IR, green; Q^2^ = 0.57). (**D**) Bar chart of log2 (fold change) for plasma biochemical features identified in the OPLS-DA model, classifying children with AR and IR children. (**E**) OPLS-DA cross-validated scores for the 24 h collected urinary ^1^H NMR metabolomes of children with AR and AR-C (Q^2^ = 0.162). (**F**) Bar chart of log2 (fold change) for 24 h collected urinary metabolites integrated from the ^1^H NMR spectra identified in the OPLS-DA model, classifying children with AR and AR C children. ALPTot, total plasma alkaline phosphatase; 1,25D, 1,25-dihydroxyvitamin D; PTH, parathyroid hormone; BALP, bone alkaline phosphatase; pPhos, plasma phosphate; 25OHD, 25-hydroxyvitamin D; iFGF23, intact fibroblast growth factor 23; TMAO, trimethylamine *N*-oxide; NMND, *N*-methylnicotinamide; NMNA, *N*-methylnicotinic acid.

OPLS models were created to determine a collection of plasma biochemical characteristics associated with disease presentation. Differences in biochemical metrics were observed between the children with AR and healthy controls (*p* = 0.001; Q^2^ = 0.68; Figure 2A,B), where children with AR had greater plasma ALPTot, BALPPTH, and 1,25(OH)_2_D compared to the controls. Conversely, children with AR had lower plasma iFGF23, 25OHD, and phosphate. Similarly, a significant model was observed, classifying the biochemical metrics of children with AR compared with those with IR (*p* = 0.001; Q^2^ = 0.57; Figure 2C,D). Six biochemical features were identified, all of which overlapped with the features discriminating between AR and AR-C. In contrast, no significant model was observed comparing the biochemical metrics of patients with IR with their healthy controls. Concentrations of plasma biochemistry features by group are documented in Supplementary Table S3.

### 3.4. Children with Active Rickets Are Metabolically Distinct from Matched Controls

To determine whether children diagnosed with rickets, either in AR or IR form, were metabolically distinct from matched control children, multivariate OPLS-DA models were generated to classify the respective metabolomes by disease group.

A significant OPLS-DA model was observed when comparing the 24 h urinary metabolic profiles of patients with AR from their matched controls (*p* = 0.02, Q^2^ = 0.162; Figure 2E,F). From this model, the urinary excretion of 13 metabolites was identified to vary in association with the presentation of active rickets. Children with AR had lower urinary β-aminoisobutyrate, alanine, dimethylamine, sucrose, 4-hydroxyphenylacetate, histidine, trimethylamine (TMAO), *N*-methylnicotinamide (NMND), and *N*-methylnicotinic acid (NMNA), as well as increased hypoxanthine, taurine, 4-hydroxyhippurate, and formate compared to matched controls. Of these, the altered excretion of hypoxanthine (β = −1.97 × 10^10^; 95% CI: −3.31 × 10^10^, −8.04 × 10^9^), 4-hydroxyphenylacetate (β = 4.50 × 10^10^; 95% CI: 1.32 × 10^10^, 7.69 × 10^10^), and NMND (β = 1.47 × 10^11^; 95% CI: 1.68 × 10^10^, 2.76 × 10^11^) remained significant in univariate regression models adjusted for age, sex, and WAZ (Supplementary Table S4).

No significant models were detected between the two-hour urinary, or plasma metabolomes of children with AR compared to matched healthy controls. Additionally, no significant models were observed between the urinary or plasma metabolomes of children with IR and matched controls, or between children with AR versus children with IR, or when any dataset was stratified by sex.

### 3.5. Integration of Clinical and Nutritional Data with the Metabolome

**Figure 3:**
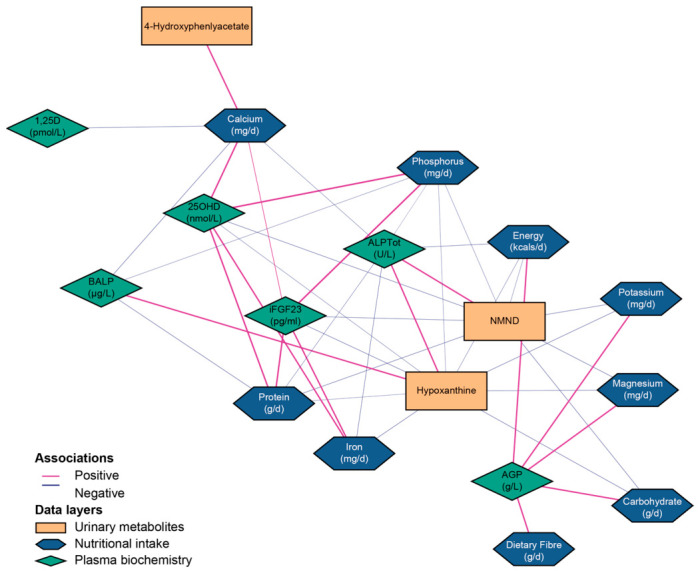
Integration identified associations between nutrition intake, plasma biochemistry and urinary metabolome. Network visualization displaying strong pairwise correlations (*r* > 0.6) identified by the Data Integration Analysis for Biomarker discovery using Latent components (DIABLO) multi-block integration model. This model highlights cross-dataset relationships that classify children with rickets (AR) from healthy matched controls. Nodes (shapes) represent variables from three data layers (24 h collected urinary metabolites integrated from ^1^H NMR spectra, orange rectangle; nutritional intake, blue hexagon; plasma biochemistry, green diamonds). Edges (connecting lines) represent the strength and direction of the association between these selected variables (positive, pink; negative, blue). ALPTot, total plasma alkaline phosphatase; AGP, α1-acid glycoprotein; 1,25D, 1,25-dihydroxyvitamin D; BALB, bone alkaline phosphatase; 25OHD, 25-hydroxyvitamin D; iFGF23, Intact fibroblast growth factor 23; NMND, *N*-methylnicotinamide.

To explore the functional relationships between dietary intake, systemic physiology and the urinary metabolome, integration was performed using DIABLO. Combining the three datasets led to a small reduction in the mean classification error rate of children with AR and healthy controls compared to the mean classification error rate observed in single block analysis (19.3% to 18.6%; Supplementary Table S5). The key benefit of the dataset integration was therefore in the exploration of relationships across different biological datasets. A relevance network analysis was performed to identify pairwise correlations (*r* > 0.60; Figure 3; Supplementary Table S6) between features selected from the three layers of data. A positive correlation was observed between 4-hydroxyphenylacetate, a gut-microbial co-metabolite, and dietary calcium intake, independently of the intake of other macronutrients. Conversely, urinary NMND and hypoxanthine were negatively correlated with a cluster of macronutrients (energy, protein, carbohydrate, magnesium, potassium, phosphorus). Dietary calcium intake was also negatively associated with plasma 1,25(OH)_2_D concentration.

Following the growth deficits observed in children with AR and IR compared to healthy controls, OPLS models were constructed to explore the metabolic variation associated with anthropometry. As discussed, children with AR and IR were shorter (length, demi-span and sitting height) than their matched controls, and additionally those with AR were lighter. No significant associations were observed between the metabolomic profiles and anthropometric measures (WAZ, HAZ, length (cm), sitting height (cm), demi span (cm), MUAC (mm), wrist width or circumference (mm)) in children with AR or IR.

### 3.6. Influence of Secondary Hyperparathyroidism on the Urinary Metabolome

Given the significant elevation of PTH in children with AR, the relationship between PTH and the 13 identified urinary metabolites was investigated within the AR group. Linear regression revealed no significant associations after Benjamini–Hochberg adjustment for multiple comparisons (*q* < 0.05; Supplementary Table S7). Although there were raw associations for formate and 4-hydroxyphenylacetate (*p* < 0.05), these were not maintained following adjustment and both exhibited very low R^2^ values (0.04, and 0.05 respectively).

## 4. Discussion

Despite the hot and tropical climate present throughout the year in Bangladesh, childhood rickets still presents a public health concern [7]. This study identified novel insights into the metabolic phenotype of nutritional rickets, where the excretion of 13 urinary metabolites varied over 24 h with active disease presentation compared to matched controls. Previous work has established a role for chronic dietary calcium deficiency in the development of nutritional rickets [1,2,3,4,5,6]; however, the broader metabolic derangements associated with the condition remain unexplored.

Among the altered metabolites, the excretion of β-aminoisobutyrate, a muscle-secreted factor derived from the catabolism of thymine or valine, was reduced in children with AR compared to controls in this study. This metabolite has been associated with improved bone mineral density [29,30] and protection of osteocytes from oxidative stress and has been proposed as a biomarker for osteoporosis [31,32]. The reduction observed here may reflect a direct effect of calcium deficiency on its production, or an indirect consequence of reduced muscle mass. Muscle deficits are plausible in this cohort, given the reduced height, demi-span and trend towards MUAC between those with AR and their controls, as well as the literature reporting muscle weakness in rickets and prior observations of reduced muscle force in animal models of poor bone mineralization [33,34]. These findings support the concept of musculoskeletal crosstalk and possibly highlight a role for muscle-derived metabolites in rickets’ pathophysiology.

Children with AR were observed to excrete lower amounts of NMND compared to matched controls. Urinary NMND is a biomarker for nicotinamide availability; nicotinamide is methylated and excreted as NMND when there is a surplus of this molecule. NAD^+^ metabolism has been linked to improved bone health, primarily via the actions of sirtuins and their regulation of oxidative stress and osteoblast activity [35,36,37,38,39,40,41,42]. The reduction in urinary NMND in children with AR may suggest impaired NAD^+^ generation, with potential downstream effects on bone cell function and growth plate maintenance. This aligns with findings in murine models demonstrating skeletal abnormalities following NAD^+^ depletion [42] and supports the further exploration of nicotinamide metabolism as a contributor to bone health in these settings.

Two metabolites identified from the multivariate models also associated with disease presentation in univariate analysis. Of these, hypoxanthine, a final ATP degradation product, was elevated in the AR children. This contrasts with the literature, where elevated hypoxanthine following L-arginine supplementation improved bone health through its anti-inflammatory effects [43]. Hypoxanthine can also be produced by intestinal bacteria from the breakdown of dietary xanthine. Such activity can stress the bioavailability of xanthine, required for purine/ATP synthesis. Interestingly, the excretion of 4-hydroxyphenylacetate, a gut microbial metabolite, was reduced with nutritional rickets [44,45]. The integration analysis identified a positive association between this metabolite and dietary calcium intake, independent of other macronutrients. 4-Hydroxyphenylacetate has not been associated with bone health previously, but various studies have demonstrated relationships between the gut microbiome and bone health [46]. This includes roles in the regulation of bone remodelling [47], skeletal development [48], and calcium absorption [49]. Additionally, prebiotic supplementation has been shown to increase calcium absorption and bone mineral density in murine models [50]. While gut microbial changes have not previously been related to nutritional rickets, this suggests that the gut microbiome may play a role in the pathogenesis of the condition and warrants further investigation.

One previous study examined the urinary metabolic profiles of Chinese children with nutritional rickets. Here, the authors identified 31 biomarkers separating children presenting with nutritional rickets from healthy controls [19]. Additionally, calcium deficiency has been investigated in the urinary and plasma metabolomes of rodents [17]. In these previous analyses, urinary taurine excretion was reduced with nutritional rickets or calcium deficiency. In contrast, urinary taurine excretion was increased in individuals with AR in this study. Taurine is involved in calcium ion transport and bone metabolism [51,52]. Due to the complex nature of metabolic pathways, alternate feedback loops or context-specific variation may explain the opposing trends observed in this metabolite.

This study confirmed the nutritional components and biochemical patterns previously observed in studies of nutritional rickets, including the low dietary intake of calcium [1,2,3,4,5,6] and phosphorus, and altered plasma 1,25(OH)_2_D, PTH and phosphate [2,3]. The most prominent biochemical shifts associated with AR in this analysis were increased plasma alkaline phosphatase and decreased iFGF23. The network analysis confirmed the expected inverse physiological correlation between dietary calcium and plasma 1,25(OH)_2_D. However, in this cohort there were no observable metabolic signatures of these individual factors in the plasma or urinary metabolomes.

Clinically, children with IR represent an intermediatory stage characterized by the resolution of the acute radiological and biochemical signatures whilst skeletal deformities remain. In this analysis, they exhibited similar metabolic profiles to their healthy controls, reflecting this clinical recovery. Despite this, the IR group also lacked statistically significant differences from the AR group in their plasma and urinary metabolomic profiles. This overlap may suggest that while IR individuals have moved towards a healthy phenotype, their metabolic recovery may be incomplete; however, these findings may also reflect a lack of power to distinguish metabolic differences.

The strengths of this study included the study design, where cases were matched with local healthy controls based on sex, age and village, as well as the integration of metabolomic, biochemical, and nutritional datasets to provide a comprehensive overview of the pathology. The limitations of this study included the relatively low sample numbers (AR *n* = 24, AR-C *n* = 23, IR *n* = 36, IR-C *n* = 35), particularly once children with active rickets were stratified from those with non-active rickets. These numbers were further reduced for the plasma samples due to residual sample volume constraints and quality control exclusions. However, attrition analysis demonstrated that missing data in the plasma samples did not introduce systematic selection bias across the cohort. The wide age range is another limitation, and stratifying individuals into narrower age brackets was not possible due to the limited sample size. The previous Chinese study (*n* = 130) was able to group children into tighter age ranges (6–12, 13–24, and 25–38 months) and may have contributed to the identification of a greater number of metabolic differences [19]. It is therefore possible that the negative findings may also reflect a lack of statistical power rather than absence of metabolic variation.

## 5. Conclusions

This study demonstrated a distinct metabolic profile associated with the presentation of active nutritional rickets and identified several novel and biologically relevant metabolites that may contribute to the presentation of disease. This included metabolites related to muscle activity, NAD^+^ and microbial metabolism. Such findings provide new understanding of the possible metabolic consequences of chronic calcium deficiency in the context of poor vitamin D status. These exploratory data highlight novel metabolic pathways that warrant further targeted investigation to fully understand their potential role in the pathophysiology of nutritional rickets. Future studies should also be carried out in larger cohorts to verify these metabolic profiles in other geographic regions and bone diseases.

## Figures and Tables

**Table 1:** Age and anthropometry of participants by group.

	Active Rickets (AR)			Inactive Rickets (IR)			
Variable	Cases (*n* = 24)	Controls (*n* = 23)	*p*	Cases (*n* = 36)	Controls (*n* = 35)	*p*	AR vs. IR *p*
Age (years)	2.98 (1.19)	3.40 (1.31)	0.26	3.39 (1.87)	3.75 (1.86)	0.42	0.30
Weight (kg)	9.65 (1.74)	11.44 (2.54)	0.01	11.36 (2.56)	12.6 (3.46)	0.10	<0.01
Height (cm)	78.9 (7.38)	90.1 (9.62)	<0.01	86.9 (9.91)	93.3 (12.9)	0.02	<0.01
WAZ	−2.99 (1.10)	−2.19 (1.09)	0.02	−2.22 (0.98)	−1.95 (1.14)	0.29	0.01
HAZ	−4.18 (1.50)	−1.97 (1.19)	<0.01	−2.60 (1.64)	−1.82 (1.39)	0.03	<0.01
MUAC (mm)	140 (7.41)	145 (10.2)	0.09	145 (9.14)	148 (11.2)	0.25	0.03
Sitting height (cm)	46.8 (3.55)	52.0 (7.80)	0.01	50.3 (4.88)	53.8 (6.17)	0.01	<0.01
Demi-span (cm)	37.0 (4.04)	40.6 (4.52)	0.01	40.5 (4.78)	43.0 (5.81)	0.05	<0.01
Thacher score	6.21 (3.51)			0.12 (0.33)			

Data described as mean (standard deviation). *p* values demonstrate significance of the difference between the cases and their matched controls for each group unless stated otherwise. WAZ, weight-for-age Z-score; HAZ, height-for-age Z-score; MUAC; mid-upper arm circumference.

## Data Availability

Data from the Bangladesh Rickets Project are archived with UKRI-MRC. The procedure for requesting data access can be found at https://www.ukri.org/publications/access-to-data-from-the-mrc-science-archive-application-form/ (accessed on 7 May 2026).

## Supplementary Materials

The following supporting information can be downloaded at: https://www.mdpi.com/article/10.3390/nu18101580/s1, Supplementary Table S1: Differences in anthropometric and demographic characteristics of those with plasma metabolomic data and those without by study group. Supplementary Table S2: Dietary intake of macro- and micronutrients by study group. Supplementary Table S3: Plasma biochemistry by study group. Supplementary Table S4: Linear regression of urinary metabolites extracted from PLS model classifying children with active rickets (AR) from healthy matched controls (AR C) following correction for covariates. Supplementary Table S5: Classification error in DIABLO (Data Integration Analysis for Biomarker discovery using Latent Components) classifying children with active rickets (AR) vs. matched controls in single and multiblock analysis. Supplementary Table S6: DIABLO (Data Integration Analysis for Biomarker discovery using Latent Components) network analysis classifying children with active rickets (AR) from matched controls (*r* > 0.6). Supplementary Table S7: Linear regression of urinary metabolites against plasma parathyroid hormone in children with active rickets (AR) following correction for covariates.
